# Inactivation of foodborne viruses by novel organic peroxyacid-based disinfectants

**DOI:** 10.3389/fmicb.2023.1187142

**Published:** 2023-05-11

**Authors:** Simon Bouchard, Teresa Paniconi, Éric Jubinville, Valérie Goulet-Beaulieu, Coralie Goetz, Patrick Marchand, Julie Jean

**Affiliations:** ^1^Département des Sciences des Aliments, Institut sur la Nutrition et les Aliments Fonctionnels (INAF), Université Laval, Québec, QC, Canada; ^2^Groupe Sani Marc, Victoriaville, QC, Canada

**Keywords:** peroxyacids, inactivation, norovirus, hepatitis A virus, hepatitis E virus

## Abstract

Viruses are responsible for most enteric foodborne illnesses worldwide. The foods most frequently involved are fresh fruits and vegetables since they undergo little or no processing. Washing with a chemical disinfectant is a convenient way of inactivating viruses on foods. Peracetic acid, widely used as a disinfectant in the food industry, has the drawback of leaving a strong odor and is ineffective alone against some foodborne viruses. In this study, four disinfectants, namely per levulinic acid with or without sodium dodecyl sulfate, peracetic acid and a commercial peracetic acid-based disinfectant were tested on murine norovirus 1 (MNV-1), hepatitis A virus (HAV), and hepatitis E virus (HEV). Disinfectant concentrations were 50, 80, 250, 500, and 1000 mg l^–1^ and contact times were 0.5, 1, 5, and 10 min. Under these conditions, per levulinic acid supplemented with 1% SDS reduced MNV-1 infectious titer by 3 log cycles vs. 2.24 log cycles by peracetic acid within 0.5 min. On stainless steel at 80 ppm, only peracetic acid produced 3-log reductions within 0.5 min. None of these peroxyacids was able to reduce infectious titers of HAV or HEV by even 2 log cycles at any concentration or time-tested. This study will guide the development of new chemical formulas that will be more effective against major foodborne viruses and will have less impact on food quality and the environment.

## 1. Introduction

Foodborne illness affects nearly 600 million people worldwide each year (World Health Organization [WHO], 2019). In Canada, this number reaches 4 million (Thomas et al., 2013) and represents up to 11,500 hospitalizations including 238 deaths in a single year (Thomas et al., 2015). The direct cost of gastrointestinal illnesses to the Canadian averages $115 per capita (Majowicz et al., 2006). A major cause of gastrointestinal disease is viruses. Human norovirus (HuNoV) and hepatitis A virus (HAV) are among the common infectious viral agents in Canada, where HuNoV is responsible for over 60% of foodborne illnesses and HAV infections number about 270 per year (Thomas et al., 2013). Other viruses that are considered emerging, such as hepatitis E (HEV), could become a food safety issue (Aspinall et al., 2017).

The prevalence of these viruses is a significant concern in the food sector, since very small numbers of viral particles are sufficient to cause infection (Ciofi-Silva et al., 2019). For example, only 18 virus particles are needed to cause HuNoV G1.1 infection 50% of the time (Teunis et al., 2008). They can persist for many weeks on surfaces such as stainless steel or foods like berries (Leblanc et al., 2019). Indeed, the irregular surface of certain small fruits, such as strawberries will exacerbate the adhesion of viruses and their persistence (Bozkurt et al., 2021). Monitoring, detecting and inactivating viruses should be essential activities to ensure food safety (Manuel and Jaykus, 2014). Means of inactivation are mainly physical and/or chemical, but a new category of chemicals of natural origin is gaining favor, for example extracts of berries (primarily cranberry and blueberry) or essential oils (Wiggienton and Kohn, 2012; Bernier et al., 2021). Due to their ease of use and low cost, chemical disinfectants are often the ideal choice for inactivating foodborne viruses (D’Souza and Su, 2009). However, some disinfectants, for example, chlorine, an inexpensive sanitizer and one of the most widely used on fresh produce, may pose health risks (Manuel and Jaykus, 2014). In contact with organic matter, chlorine can form the carcinogen trihalomethane (Mohamadshafiee and Taghavi, 2012; Pérez-Lavalle et al., 2020), more specifically chloroform (Gomez Camponovo et al., 2014). Inadequate rinsing of chlorine can accelerate the corrosion of stainless steel food processing equipment (Rutala, 2008). The food industry is therefore searching for safer alternatives to this agent. Less reactive compounds such as peroxyacids have been considered.

Peroxyacids are obtained from the reaction of an organic acid with hydrogen peroxide (Fuzawa et al., 2020). They have strong oxidizing power and can thus damage viral capsid nucleic acids and proteins (Vimont et al., 2015). They are more antiviral than the organic acid reagent (Vimont et al., 2015) and unlike chlorine, they quickly break down to mostly water and safe by-products including the organic acid and hydrogen peroxide (Block, 2016). However, viruses can adapt in ways that reduce the effectiveness of peroxyacid disinfectants and detergents on food surfaces, for example, aggregation and increased adhesion (Mattle et al., 2011). Because focused studies remain rare, this family of chemical compounds is still barely used in the food sector as antiviral disinfectants.

Another peracid called perpropionic acid has been shown to be effective against murine norovirus 1 in suspension (Vimont et al., 2015), producing a 4-log reduction within 5 min at 50 ppm. Similar results have been obtained with peracetic acid (Vimont et al., 2015). A solution of 2% levulinic acid and 1% sodium dodecyl sulfate has been found synergic in reducing murine norovirus 1 (MNV-1) titer by 4.21 log cycles within 1 min (Cannon et al., 2012). At 0.5% each, these compounds have reduced HAV titers by 2.7 log cycles on strawberries within 2 min of contact time but MNV-1 by only 1.4 log cycles (Zhou et al., 2017). Whether converting levulinic acid to its peroxy form increases or decreases the synergism with SDS is worthy of investigation.

The aim of the present study was to analyze the virucidal potential of per levulinic acid without and with 1% SDS, peracetic acid alone, and a commercial peracetic-acid-based disinfectant containing SDS. The viruses tested were MNV-1, HAV, and HEV on stainless steel, strawberries, and blueberries. MNV-1 is often used as a proxy for HuNoV because its plaque assay is reliable and its genetics and morphological characteristics are quite similar (Wobus et al., 2004; Estes et al., 2019). This study may guide the development of commercial peroxyacid products effective against a wide range of foodborne viruses.

## 2. Materials and methods

### 2.1. Cell lines and virus

RAW 264.7 cells (ATCC^®^ TIB-71) were cultured in Dulbecco-modified Eagle’s medium (DMEM) supplemented with 10% fetal bovine serum plus 50 IU of penicillin per ml and 50 mg of streptomycin per liter (all from Wisent Inc., Canada) and were frozen at −80°C until use (Gonzalez-Hernandez et al., 2012). MNV-1 (ATCC^®^ VR-1937) was propagated in RAW 264.7 cells for 60 h at 37°C with 5% CO_2_ then released by 3 cycles of freezing and thawing. The lysate was centrifuged at 2,000 × *g* for 15 min at 4°C to pellet cell debris. The supernatant was deposited on 5 ml of 30% (w/v) of sucrose solution (sterilized by microfiltration, Fisher Scientific, USA) in extra clear Quick-Seal tubes (Beckman Coulter, USA) and ultra-centrifuged at 95,000 × *g* for 3 h at 4°C in an 41 Ti rotor (Beckman Coulter, USA). The supernatant was discarded, the pellet was re-suspended overnight in 400 μl of PBS 1X and the tube bottom was washed with 100 μl of PBS 1X (added to the suspension). The 500 μl of concentrated virus (5.1 × 10^8^ pfu/ml) was aliquoted and stored at −80°C.

FRhK-4 cells (ATCC^®^ CRL-1688) were cultured in Eagle’s minimal essential medium (Wisent Inc., Canada) supplemented with 10% fetal bovine serum plus 50 IU penicillin per ml and 50 mg of streptomycin per liter and were frozen at −80°C until use (Mbithi et al., 1991). HAV strain HM-175 (Health Canada Bureau of Microbial Hazards) was propagated in FRHK-4 cells for 8 days at 37°C with 5% CO_2_ then released by 3 cycles of freezing and thawing. The lysate was processed as described above for MNV-1 and the concentrate was stored at −80°C. The titer was 4.1 × 10^8^ pfu/ml.

A549 cells persistently infected with HEV strain 47832c (kindly provided by Dr. Reimar Johne from the German Federal Institute for Risk Assessment) were cultured in minimal essential medium supplemented with 10% fetal bovine serum, 1% non-essential amino acids, 1% L-glutamine (all from Wisent Inc., Canada) and 0.5% gentamicin (Sigma-Aldrich, Canada) as described elsewhere (Schemmerer et al., 2016) and were frozen at −80°C until use. HEV was recovered by harvesting the supernatant after 7 days of culture at 37°C with 5% CO_2_ and centrifuging at 2,000 × *g* for 15 min at 4°C. The clarified supernatant was stored at −80°C (Johne et al., 2014, 2016). The titer was 1.24 × 10^6^ genome copies/μl.

### 2.2. Peracid preparations

Peracid was prepared as described previously (Vimont et al., 2015). Basically, 1.5 volumes of glacial acid acetic (Sigma-Aldrich, Canada) or levulinic acid (product L2009, Sigma-Aldrich, Canada) was mixed with 1 volume of 30% hydrogen peroxide (Sigma-Aldrich) and 1% w/w of sulfuric acid (Thermo-Fisher Scientific, USA). The solution was kept at 30°C for 48 h and the peracid and hydrogen peroxide concentrations were determined 24 h before viral inactivation treatment by colorimetric titration with cerium sulfate IV (Thermo-Fisher Scientific, USA) and sodium thiosulfate (Acros Organics, United Kingdom). When sodium dodecyl sulfate (Sigma-Aldrich, Canada) was used, it was added just before viral inactivation treatment.

### 2.3. Viral inactivation treatments

#### 2.3.1. Inactivation in liquid medium

10 μl of the MNV-1 viral suspension was diluted 1:10 in disinfectant solution at concentrations adjusted to obtain 50, 80, 250, 500, or 1000 ppm of tested compound. Contact times were 30 s, 1, 5, and 10 min in accordance with ASTM standard test method E 1052-20 and the viral titer in absolute tested was 5.1 × 10^5^ pfu. The inactivation was stopped by diluting at 1:10 in DMEM supplemented with L-cysteine hydrochloride (product C7477, Sigma-Aldrich, Canada). Residual MNV-1 titer was determined by plaque assay as described below.

#### 2.3.2. Inactivation on stainless steel

Stainless steel test coupons 1 cm in diameter were washed once with 1% Tergazyme^®^ (Alconox, USA) solution for 5 min then rinsed three times with distilled water, autoclaved for 20 min at 121°C then exposed to UV light for 15 min on each side. The experiments were conducted in accordance with ASTM standard test method E 1053-20. Briefly, 2 μl of viral concentrate (1.2 × 10^5^ pfu of MNV-1, or 8.2 × 10^4^ pfu of HAV or 2.48 × 10^6^ genome copies) were spotted on each coupon and dried for 30 min under a laminar flow hood. The inactivation treatment consisted of adding 18 μl of peracid disinfectant to the spot then submerging the coupon in 2 ml of Earle’s balanced salt solution (EBSS, Thermo-Fisher, USA) with L-cysteine hydrochloride, adding 20 glass beads (1 mm diameter, cleaned in the same manner as the test coupons) and vortex mixing for 1 min. The residual viral titer was determined by plaque assay (MNV-1 and HAV) or PCR (HEV) as described below.

#### 2.3.3. Inactivation of MNV-1 on food matrices

Fruit purchased in a local supermarket was prepared by washing three times in distilled water and once in sterile distilled water under a laminar flow hood. Blueberries were used whole whereas strawberries were pealed to expose 1 cm^2^ of moist surface. The berries were allowed dry for 1 h then exposed to UV light for 20 min. MNV-1 at 5.1 × 10^5^ pfu in absolute was spotted (1 μl) on the prepared surface and allowed to dry for 45 min. Peracid disinfectant (9 μl) was then placed on the same spot and left for the prescribed time, after which the inactivation treatment was ended by withdrawing the disinfectant into a micropipette and washing the spot 3 times by drawing 15 times 30 μl of EBSS with a micropipette tip. The residual MNV-1 titer was determined by plaque assay as described below.

#### 2.3.4. Quantification of MNV-1 and HAV by plaque assay

RAW 264.7 cells and FRhK-4 cells were seeded at, respectively, 8.6 × 10^5^ and 1.25 × 10^5^/well in 12-well plates in their respective medium and incubated for 24 h at 37°C with 5% of CO_2_ as described previously (Mbithi et al., 1991; Gonzalez-Hernandez et al., 2012). The medium was then removed, and the cell monolayer was washed twice with 2 ml of PBS 1X, spread with 300 μl of virus sample diluted 10-fold serially in DMEM and incubated for 90 min. The viral sample was removed, and RAW 264.7 monolayers were overlaid with (per well) 3 ml of minimal essential medium containing 10% fetal bovine serum, 2% L-glutamine, 10 mM HEPES (Wisent Inc., Canada), 0.3% sodium bicarbonate (Wisent inc., Canada) and 0.8% SeaPlaque™ agarose (Lonza, Switzerland) and incubated for 48 h at 37°C with 5% CO_2_, whereas FRhK-4 monolayers were overlaid with 3 ml of minimal essential medium containing 2% fetal bovine serum, 2% L-glutamine, 1% non-essential amino acids, 10 mM HEPES, 0.113% sodium bicarbonate (Wisent Inc., Canada), 50 IU/ml penicillin and 50 μg/ml streptomycin, 0.5% magnesium chloride (Avantor Inc., USA), and 0.8% SeaPlaque™ agarose and incubated for 8 days. Both cell types were then fixed for at least 5 h with 3.7% formaldehyde (VWR International, USA) in physiological saline. Cells were then colorized for 10 min with 1% w/v crystal violet solution (Sigma-Aldrich, Canada) (Mbithi et al., 1991).

#### 2.3.5. Extraction of HEV

After inactivation of HEV, 15 μl of platinum (IV) chloride solution (50 μM, Sigma-Aldrich, Canada) were added and the suspension was held at 4°C with a constant agitation for 30 min to remove free viral RNA (Randazzo et al., 2018). Capsids were then lysed by adding 2 ml of NucliSens^®^ easyMAG™ lysis buffer (BioMérieux, France). After 10 min, capsid viral RNA was extracted using an eGENE-up system (Biomérieux), a semi-automated extraction platform. Samples were eluted in 100 μl of elution buffer and stored at −80°C. The HEV genome was amplified using the iTaq Universal probe 1-step (Bio-Rad, USA) with an ABI7500 real-time PCR system (Applied Biosystems, Thermo-Fisher Scientific, USA) using the SDSv1.4 program. The probes (Integrated DNA Technologies, USA) were used (Martin-Latil et al., 2016). The primers (Integrated DNA Technologies, USA) were used as described elsewhere (Germer et al., 2017). The master-mix consisted of 10 μl of iTaq universal probe reaction mixture (2 ×), 2.63 μl RNAase-free water, 250 nM of each primer, 250 nM of the probe, 0.5 μl of iScript advanced reverse transcriptase and 5 μl of RNA sample. Pure and 10-fold diluted RNA extracts were plated manually in 96-well PCR microplates (Axygen, USA) in duplicate. The plates were then sealed with an optical adhesive film (Applied Biosystems, USA). A negative RT-qPCR control consisting of RNAase-free water was run. Reverse transcription was performed at 50°C for 10 min followed by polymerase activation and DNA denaturation at 95°C for 3 min and 45 cycles of 95°C for 15 s and 60°C for 0.5 min. Genome copies were quantified using a dsDNA standard curve generated from a serial 10-fold dilution of dsDNA plasmid pIDTSmart-AMP (IDT, USA) bearing the target sequence and designed based on the primers and the HEV genome (ref. NC_001434.1). The standard curve was produced in triplicate.

### 2.4. Statistical analysis

MVN-1 inactivation in suspension and HAV or HEV inactivation on surface results were analyzed using a two-way ANOVA with a Tukey’s multiple comparisons test in GraphPad Prism 9 version 9.1.2. For MNV-1 inactivation on fruit and surfaces, results were analyzed using a three-way ANOVA with a Tukey’s multiple comparison test. Due to a high number of statistical comparisons in some figures, only the most relevant ones are shown and discussed. All experiments were performed in triplicate.

## 3. Results

### 3.1. Inactivation of MNV-1 in solution

**FIGURE 1:**
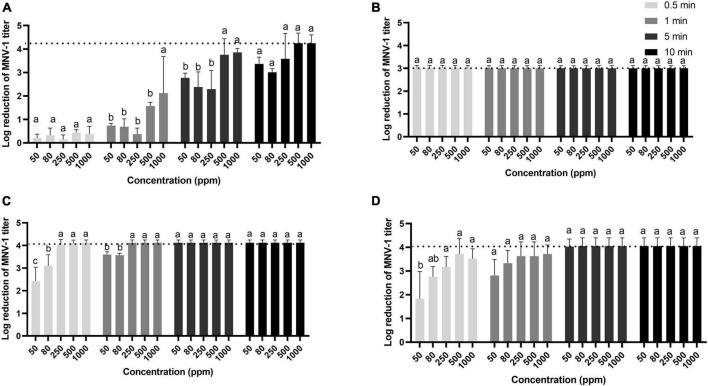
Inactivation of MNV-1 in suspension by per levulinic acid **(A)**, per levulinic acid with 1% sodium dodecyl sulfate **(B)**, peracetic acid **(C)** or a commercial peracetic-based disinfectant **(D)**. The dotted line represents the limit of plaque-forming unit detection due to the dilution required to avoid cytotoxic effects. Values are means of three replications. Error bars represent standard deviation. Treatments with different letters differ significantly (*p* < 0.05).

Per levulinic acid was the least effective of the four disinfectants tested against MNV-1 in suspension (Figure 1). However, its antiviral effect was greatly influenced by time and less by concentration, reducing the viral titer by at least 3 log cycles after 10 min at all concentrations tested. The per levulinic acid/SDS combination was the most effective, producing the 3-log reduction even under the minimal treatment condition (50 ppm for 30 s). Because of cytotoxicity, it was not possible to observe how much greater the reductions might have been. Peracetic acid and the commercial disinfectant based on peracetic acid gave similar results, a 3-log reduction in 1 min at 80 ppm and 4 log cycles in 5 min at 50 ppm, respectively (Figure 1).

### 3.2. Inactivation of MNV-1 on surfaces

**FIGURE 2:**
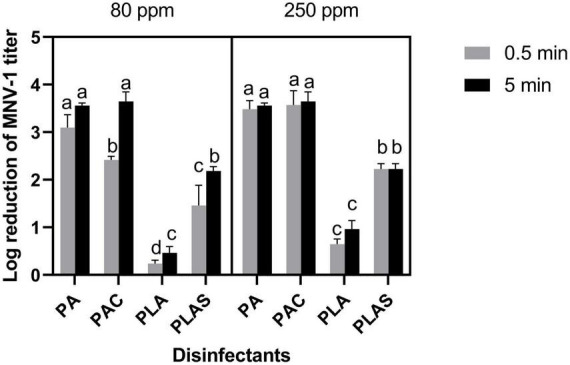
Inactivation of MNV-1 on stainless steel by peracetic acid (PA), a commercial peracetic-acid-based disinfectant (PAC), per levulinic acid (PLA) or per levulinic acid with 1% sodium dodecyl sulfate (PLAS). Values are means of three repetitions. Error bars represent standard deviation. Treatments with different letters differ significantly (*p* < 0.05).

On stainless steel, per levulinic acid was ineffective at all concentrations and contact times tested, giving <1 log reductions of MNV-1 titer. Per levulinic acid combined with SDS reduced the titer by 2.19 ± 0.09 log cycles in 5 min at 80 ppm and by 2.22 ± 0.11 log cycles in 30 s at 250 ppm. However, because of the cytotoxicity of this agent, the maximal reduction in titer by this treatment could not be measured. Thirty seconds at 80 ppm was ineffective (1.45 ± 0.43 log reduction). These conditions were sufficient for peracetic acid to reduce the titer by 3.10 ± 0.27 log cycles whereas the peracetic-based commercial disinfectant was slightly less effective (2.41 ± 0.07 log cycles). However, both gave 3.5 log cycle reductions of MNV-1 titer after 5 min at all concentrations tested (Figure 2).

### 3.3. Inactivation of MNV-1 on berries

**FIGURE 3:**
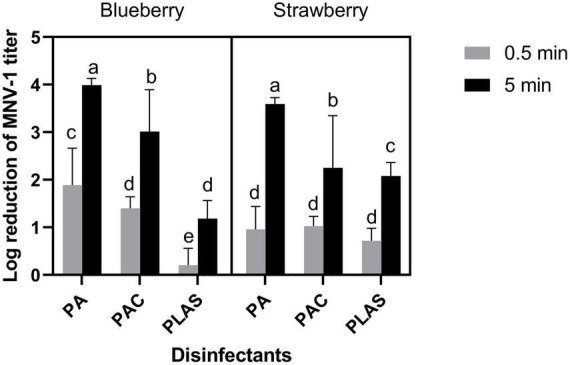
Inactivation of MNV-1 on fresh blueberry and strawberry by peracetic acid (PA), a commercial peracetic-acid-based disinfectant (PAC) or per levulinic acid with 1% of sodium dodecyl sulfate (PLAS). Values are means of three repetitions. Error bars represent standard deviation. Treatments with different letters differ significantly (*p* < 0.05).

Because of its weak antiviral activity on stainless steel, per levulinic acid alone was not tested on berries. Overall, 0.5 min of contact time was insufficient for any of the other agents to inactivate MNV-1 appreciably on either fruit (Figure 3). However, after 5 min, peracetic acid at 80 ppm reduced the titer by 3.99 ± 0.14 log cycles on blueberries and by 3.59 ± 0.13 log cycles on strawberries. The corresponding log reductions by peracetic acid disinfectant were 3.01 ± 0.88 and 2.25 ± 1.09 and by per levulinic acid/SDS 1.18 ± 0.38 and 2.08 ± 0.29.

### 3.4. Inactivation of HAV and HEV on surfaces

**FIGURE 4:**
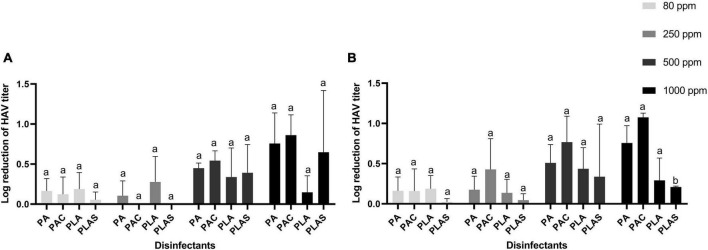
Inactivation of HAV after 0.5 min **(A)** or 1 min **(B)** on stainless steel with peracetic acid (PA), a commercial peracetic-acid-based disinfectant (PAC), per levulinic acid (PLA) or per levulinic acid with 1% sodium dodecyl sulfate (PLAS). Values are means of three repetitions. Error bars represent standard deviation. Treatments with different letters differ significantly (*p* < 0.05).

**FIGURE 5:**
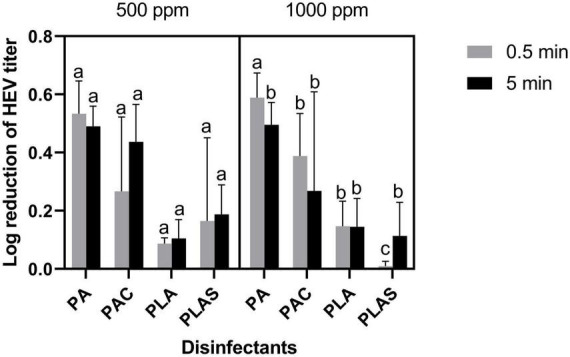
Inactivation of HEV on stainless steel with peracetic acid (PA), a commercial peracetic acid-based disinfectant (PAC), per levulinic acid (PLA) or perlevulinic acid with 1% sodium dodecyl sulfate (PLAS). Values are means of three repetitions. Error bars represent standard deviation. Treatments with different letters differ significantly (*p* < 0.05).

None of the agents tested as antiviral disinfectants had any appreciable effect on HAV (Figure 4) or HEV (Figure 5). The greatest log reduction of HAV plaque-forming titer was 1.17 by 1000 ppm of the commercial peracetic-acid-based disinfectant, whereas HEV was for all practical purposes resistant to all four agents at this concentration even after 5 min of contact.

## 4. Discussion

Several chemical disinfectants are well established in the food sector. However, these may not be antiviral at concentrations safe for use in foods or in the workplace. The use of peroxyacid-based disinfectants is innovative and could reduce the negative impact of efforts to suppress foodborne viruses. Peracetic acid at least than 100 ppm has been shown to inactivate Gram-positive and Gram-negative bacteria, yeasts, and molds in suspension within 5 min of contact time (Disinfection Sterilization Guidelines et al., 2019). Peracetic acid has been shown to be effective against several viruses (D’Souza and Su, 2009; Vimont et al., 2015; Fuzawa et al., 2020). At 80 ppm for 1 min, it has been shown to reduce MNV-1 plaque-forming titer by 3 log cycles (Min et al., 2023). It has been found much less effective (1.24 log reduction) for inactivating HAV, even at 2500 ppm for 10 min (Song et al., 2022). Other peroxyacids such as perlactic acid (250 ppm) or perpropionic acid (50 ppm) have shown to reduce MNV-1 titers in suspension by 4 log cycles within 5 min (Vimont et al., 2015). The FDA has not authorized more than 80 ppm of peracetic acid for washing fruits and vegetables (U.S. Food and Drug Administration, 2023a). Concentrations not less than 200 ppm and as high as 315 ppm are authorized in the USA FDA Code of Federal Regulations Title 21 section 178.1010 (c) (33) for surface washing without rinsing (U.S. Food and Drug Administration, 2023b). Few studies have examined the mechanisms by which peroxyacids inactivate foodborne viruses. One possibility is damage to viral RNA by free radicals generated the breaking of the peroxide bond (Vimont et al., 2015). At higher concentrations of peroxyacid, viral capsid proteins could also be damaged. Peroxyacetic acid also seems to cause some viruses to aggregate, which would increase their resistance to chemical treatment. The Tulane virus apparently aggregates in the presence of peracetic acid while rotavirus does not (Pérez-Lavalle et al., 2020). Although this phenomenon appears to be pH-dependent, virus isoelectric point apparently is not involved, thus making the disinfection power of peracetic acid virus-specific. These observations may explain why none of the peroxy agents tested in the present study had much effect on HAV or HEV whereas they were at least somewhat effective or very effective against MNV-1. Although the mechanism remains the subject of speculation, it has been shown elsewhere that HAV is more resistant than MNV-1 to disinfectants (Fraisse et al., 2011; Gibson and Schwab, 2011; Brié et al., 2018).

The antiviral effect of SDS with levulinic acid appears to be synergic against MNV-1 in suspension (Vimont et al., 2015). We found a similar synergism with levulinic acid in its peroxyacid form, given that SDS alone (1% for 10 min) did not reduce even by 1 log cycle the titers of HAV or MNV-1 in suspension (data not shown) and per levulinic acid alone at 50 ppm for 0.5 reduced MNV-1 titer by less than twofold (Figure 1). The combination of 1% SDS with 50 ppm of per levulinic acid also has the advantage of being odorless. A 3-log reduction of MNV-1 titer has been obtained in 1 min with 2% SDS and 5% levulinic acid (Vimont et al., 2015) and numbers of infectious HAV particles on strawberries reportedly fall by 2.7 log cycles after 2 min of contact with a disinfectant containing 0.5% levulinic acid and 0.5% SDS (Zhou et al., 2017). The mechanism of synergic action of levulinic acid with SDS remains unclear, although it appears to be pH dependent (Vimont et al., 2015). SDS is known to affect enveloped virus like herpes simplex virus and human immunodeficiency virus (Piret et al., 2000) and naked viruses like the human papillomaviruses (Howett et al., 1999). It likely works by destabilizing the envelope and/or by denaturing the viral capsid protein.

The troubling resistance of the emergent foodborne and zoonotic virus HEV to the disinfectant agents was not unexpected. It has been shown that naked and quasi-enveloped HEV both are resistant to alcohol-based disinfectant but less so to phosphoric acid (Behrendt et al., 2022) or to chlorine in drinking water, where infectious titers may fall 4 log cycles after 15 min of exposure to 3 mg/l. Molecular detection (RT-qPCR) allows quantification of viral RNA but without distinction between infectious and non-infectious virions. An intercalating agent such as PtCl_4_ or propidium monoazide can be used to reduce the amount of inactivated virus co-detected (Randazzo et al., 2018). Viral plaque assay is the most suitable method of quantifying residual infectious virus following an inactivation treatment. However, the cells used for this purpose may be sensitive to the chemicals being tested and require dilution of the sample beyond the limit of meaningful detection of virus. Unfortunately, per levulinic acid/SDS was toxic to RAW 264.7 cells, which limited the readable results to the last three 10-fold dilutions, at which the apparent reduction in infectious viral titer was 3 log cycles. In addition, this effect was exacerbated in the experiments on viral inactivation on surfaces, due to the greater difficulty of recovering the virions using a pipette.

Since peracetic acid and per levulinic acid gave some interesting results, the antiviral potential of other peracids such as perpropionic, percitric or perlactic acid in combination with a surfactant such as SDS should also be studied. The creation of novel peracids with unusual organic acids should also be investigated in addition to possible synergic effects with other surfactants such as triton. However, there are still some analyses to be carried out, such as the evaluation of the stability of per levulinic acid, its toxicity and its persistence. The development of a safer chemical antiviral formula will help reduce industry costs associated with food recall, ensure food safety for consumers, and reduce the harmful effects of certain chemical disinfectants in the environment.

## Data availability statement

The raw data supporting the conclusions of this article will be made available by the authors, without undue reservation.

## Author contributions

SB, CG, ÉJ, VG-B, and JJ: conceptualization. SB: methodology, investigation, resources, and preparation/writing of the original draft. ÉJ, CG, VG-B, and JJ: validation. SB and ÉJ: formal analysis. JJ: data curation and funding acquisition. SB, ÉJ, VG-B, PM, and JJ: writing, review, and editing. ÉJ, VG-B, and JJ: supervision. ÉJ, VG-B, PM, and JJ: project administration. All authors contributed to the article and approved the submitted version.
